# Lymphocystis viral disease impacts the diversity and functional profiles of the skin microbiome in gilthead seabream

**DOI:** 10.3389/fmicb.2024.1470572

**Published:** 2024-10-21

**Authors:** Raquel Xavier, Marcos Pérez-Losada, Sofia Marques Silva, Marilia Lino, Maria João Faleiro, Paula Canada

**Affiliations:** ^1^CIBIO, Centro de Investigação em Biodiversidade e Recursos Genéticos, InBIO Laboratório Associado, Universidade do Porto, Porto, Portugal; ^2^BIOPOLIS Program in Genomics, Biodiversity and Land Planning, CIBIO, Vairão, Portugal; ^3^Computational Biology Institute, Department of Biostatistics and Bioinformatics, Milken Institute School of Public Health, The George Washington University, Washington, DC, United States; ^4^Department of Computer Sciences, Faculty of Sciences, University of Porto, Porto, Portugal; ^5^Marisland–Madeira Mariculture, Lda., Funchal, Madeira; ^6^CIIMAR/CIMAR-LA–Centro Interdisciplinar de Investigação Marinha e Ambiental, Universidade do Porto, Matosinhos, Portugal

**Keywords:** bacteria, aquaculture, dysbiosis, *Sparus aurata*, *Tenacibaculum maritimum*

## Abstract

Lymphocystis viral disease (LVD) is a highly transmissible disease known to affect multiple fishes worldwide. Although this disease is usually benign, mortalities can occur in cases where infection is severe or secondary infection with bacterial pathogens and parasites occur. However, little is known about the bacterial dynamics of fish with LVD or what bacterial pathogens may be responsible for secondary infections. Here we assessed the effects of LVD on the skin microbiome of gilthead seabream by comparing 30 symptomatic, asymptomatic and recovered (three weeks after infection) fish using 16S rRNA high-throughput sequencing. Our results show that LVD is associated with significant changes in microbiome structure and function. Importantly, fish pathogens like *Tenacibaculum maritimum* and some *Vibrio* species increased their abundance. Moreover, microbial metabolic activities of the commensal microbiota that may confer some protection to fish were suppressed in diseased fish. After reducing fish cage density to treat symptoms and three weeks of recovery, the abundance of pathogens was significantly reduced and microbiome functionality was recovered, although community structure remained significantly different. These results show that LVD can severely disrupt the bacterial communities of the skin of the gilthead seabream, leading to an increase in bacterial pathogens responsible for relevant diseases in gilthead seabream farms.

## 1 Introduction

Lymphocystis viral disease (LVD) is a chronic, self-limited but highly transmissible disease, known to affect multiple fishes worldwide (Borrego et al., 2017). Symptoms include the appearance of external tumor-like nodules that affect fish growth and can last weeks or even months (e.g. Basurco et al., 1990). Although usually benign, this disease is systemic affecting multiple internal organs as well (Valverde et al., 2017), with fish mortalities being sometimes reported and associated with gill functioning impairment, compromised swimming ability and with secondary bacterial and parasitic infections (Moate et al., 1992; Haddad-Boubaker et al., 2013; Borrego et al., 2017). Disease triggers appear to be connected with environmental cues (e.g., temperatures > 20 C and pollution) and stress associated with intensive aquaculture practices, including high fish density and handling (reviewed by Borrego et al., 2017).

Although LVD etiology is classically associated with the iridovirus LCDV, in the gilthead seabream (*Sparus aurata*, L.) the development of symptoms is associated with the co-occurrence of LCDV-Sa l with either of two other viruses: *Sparus aurata* papillomavirus 1 (SaPV1) and polyomavirus 1 (SaPyV1) (López-Bueno et al., 2016). Indeed, a follow up study uncovered that from 59 LVD symptomatic fish infected with LCDV-Sa, almost all were also infected with either SaPV1 or SaPyV1 (98%), and a high proportion carried the three viruses (78%). In contrast, in 71 asymptomatic fish, fewer were infected with multiple viruses (25% carried at least two viruses and only 1.4% carried all three viruses), although many were carriers of the LCDV-Sa (75%) (Labella et al., 2019). This results suggest a relation between a polyviral etiology for LVD and the manifestation of symptoms. Interestingly, the infectability of LCDV in water also seems to depend on other planktonic microbiota; this was shown for the LCDV strain Leetown NFH (ATCC VR-342), which decayed rapidly (15 days at 22 C) in untreated seawater, whereas it remained detectable in treated water (autoclaved or filtered) after 6 months (Leiva-Rebollo et al., 2020).

Associations between viral diseases in fish and secondary bacterial infections are common (García-Rosado et al., 2007), and although many reports mention the possibility of bacterial secondary infection aggravating the symptoms of fish with LVD, very few have reported meaningful bacteriological results (e.g. Dezfuli et al., 2012; Haddad-Boubaker et al., 2013), with details on the taxonomy of such bacteria still largely lacking. Pathogenic species of *Vibrio* and *Photobacterium damselae* are usually reported in diseased sparids in association with viral nervous necrosis and viral hemorrhagic septicemia (García-Rosado et al., 2007). Results from a handful of studies using high-throughput sequencing to characterize the bacteriome of fish suffering from viral diseases suggest that dysbiosis of the fish bacteriome caused by viruses is frequent and leads to significant changes in bacterial community structure and increases in the abundance of potential pathogens (Xavier et al., 2024).

Previous studies on the skin microbiome of healthy gilthead seabream have shown high inter-individual variability (Chiarello et al., 2015); that fish microbiotas naturally harbor potential pathogenic species (Rosado et al., 2019) and that their diversity varies with age (Rosado et al., 2021). The reported effects of bacterial disease and skin injuries on the skin of the gilthead seabream include changes in community structure (Rosado et al., 2023) that may encompass decreases in beneficial bacteria (Tapia-Paniagua et al., 2018) and increases in potentially pathogenic species (Tapia-Paniagua et al., 2018; Rosado et al., 2023). Additionally, the protective role of skin microbiota can also be affected, namely through a reduction in the number of enriched pathways related to detoxification (Rosado et al., 2023).

In the present study, we have used 16S rRNA high-throughput sequencing to study the bacterial microbiota of a population of farmed gilthead seabream showing symptoms of LVD as well as asymptomatic fish collected from the same rearing tank. Given that asymptomatic seabream can be carriers of chronic but sub-clinical infections by LCDV, we aimed to understand to what extent this virus impacts the bacteriome of fish showing disease symptoms and identify the pathogens that more likely contribute to secondary bacterial infections. Additionally, we have also sampled the same fish population three weeks after the onset of infection, when no fish showed symptoms of LVD, to ascertain whether potential changes persisted after recovery.

## 2 Materials and methods

### 2.1 Ethics statement

All procedures implying fish handling and sampling were performed by a trained scientist and following the European Directive 2010/63/EU of European Parliament and of the Council of European Union on the protection of animals used for scientific purposes.

### 2.2 Experimental design and sample collection

The studied fish belonged to a batch of gilthead seabream juveniles being grown in a single 10 m^3^ fiberglass tank (tank 1) in a flowthrough system at the Mariculture Centre of Calheta (Madeira Island, Portugal), at an average temperature of 22.1 ± 0.4 C, dissolved oxygen (DO) above 95% of saturation and salinity at 39 ppt. Fish were fed a commercial diet from Aquasoja, NeoGold NEO1.5, Moisture 8%; Crude Protein 55%; Crude Fat 16%, Ash 10%; Crude Fibers 1%; Phosphorus 1.4%; Digestible Energy 18.3 kJ/g; DP/DE 26 mg/kJ. In July 25^th^ 2020 some fish developed lymphocyst and five days later, about half of the fish showed lymphocysts lesions in their skin. The first sampling time point was that same day (July 30^th^). Ten fish showing lymphocysts lesions in their skin were collected and identified as *symptomatic* (13.1 ± 3.8 g, 9.9 ± 1.1 cm) and ten fish showing no signs of disease were collected and identified as *asymptomatic* (15.6 ± 5.9 g, 10.6 ± 0.7 cm). Half of the tank population was moved the next day to a new tank (tank 2), with no discrimination between symptomatic and asymptomatic fish, and density was decreased by 2-fold to ∼6 Kg/m^3^. In the following three weeks, the water temperature averaged 23.1 ± 0.4°C and tank’s DO was kept over 90%, with most of the fish looking healthy at the end of three weeks. By then, ten fish showing no signs of disease were collected from tank 1 and identified as *recovery* (33.4 ± 3.8 g, 12.9 ± 0.4 cm).

To avoid any possible effects of anaesthesia on skin microbiome disruption, fish were killed by a blow to the head followed by decapitation –this is a method conditionally accepted by the European Directive PE-CONS 37/10 and the Portuguese Legislation (December 113/2013). Sampling took place in a sterile environment. Skin microbial samples were collected using tubed sterile dry swabs (Medical Wire and Equipment, UK) and by swabbing several times along the right upper lateral part of the fish from head to tail. Fish were individually weighed and measured. Swabs were immediately stored at −20°C until transported on dry ice to the CIBIO-InBIO laboratory by airmail, where they were kept at −80°C until further processing.

### 2.3 DNA extraction, library preparation and sequencing

DNA from swabs was extracted using the MO BIO Power Soil DNA Isolation Kit (QIAGEN, Hilden, Germany). Purified DNA was sent to the Centre for Microbial Systems of the University of Michigan Medical School (USA) to be amplified and sequenced. The V4 region of the 16S rRNA gene (∼ 250 bp) was amplified from each sample using the dual indexing sequencing strategy developed by Kozich et al. (2013). Each sample, four replicates of a mock community (ZymoBIOMICS Microbial Community DNA Standard, Zymo Research, USA), two extraction blanks and four PCR controls were sequenced on a single run of the Illumina MiSeq platform.

### 2.4 Bioinformatic and statistical analysis

All analysis were performed in R v4.3.1. The package DADA2 (version 1.28.0, Callahan et al., 2016) was used to denoise raw sequences. Following visual inspection of the quality of forward and reverse reads the filterAndTrim command was used, setting options TruncLen = c(200, 180) and maxEE = C(2, 2) and all remaining parameters set as defaults. In total, 1,113,742 partial 16S rRNA gene sequences were retrieved for all seabream individuals. Taxonomic inferences were done against the SILVA (138 release) reference database (Quast et al., 2013) and ASVs classified as Archaea, and those classified as bacteria but belonging to order “Chloroplast” and family “Mitochondria” were filtered from the dataset. The MIMt taxonomic database (Cabezas et al., 2023) was used to confirm taxonomic assignments of interest. Additionally, 39 ASVs present in negative controls (extraction kit and PCR) and mock communities were also removed from downstream analysis. In extraction controls, contaminants included ASVs from *Pseudomonas*, *Blautia*, *Eubacterium*, *Limosilactobacillus* and *Lawsonella*. PCR controls contained contaminants from *Pseudomonas*, *Barnesiella*, *Paucibacter* and two unclassified bacteria. ASVs of *Pseudomonas* were the most frequent contaminant (70% of contaminant ASVs in extraction controls and 50% of contaminant ASVs in PCR controls). After the removal of contaminants, nonbacterial sequences and chimaeras, 1,048 ASVs were assigned to the microbes in the skin of the seabream. From these, 461 ASVs (223,371 reads), 417 ASVs (288,271 reads) and 424 ASVs (245,180 reads) were recovered from the three fish groups: asymptomatic, symptomatic and recovery, respectively. The average number of reads per sample was 25,227 (min = 5,263 and max = 45,154; rarefaction curves in Supplementary Figure 1).

The ASV table was normalized using the negative binomial distribution (McMurdie and Holmes, 2014) and a midpoint rooted tree of ASVs was estimated using the Quantitative Insights Into Microbial Ecology 2 package (QIIME2; release 2020.11; Bolyen et al., 2019). All analyses were performed using the normalized ASV table unless stated otherwise.

#### 2.4.1 Analysis of bacterial diversity and composition

Microbial alpha-diversity was estimated at the ASV-level using Richness, Shannon diversity, and Faith’s phylogenetic (PD) diversity indices as implemented in the R packages phyloseq and picante (Kembel et al., 2010; McMurdie and Holmes, 2013). Microbiome structure (beta-diversity) was also estimated at the ASV-level using phylogenetic UniFrac (weighted and unweighted), Bray–Curtis, and the Jaccard distances in the phyloseq package (McMurdie and Holmes, 2013). Variation in microbial alpha-diversity between groups was assessed using linear models (lm) as implemented in the R stats package (R Core Team, 2012). Variation in microbial beta-diversity was assessed using permutational multivariate analysis of variance (PERMANOVA) implemented in the adonis2 function of the R vegan package (Oksanen et al., 2014), followed by the pairwise.adonis2 test when significant differences were found. Dissimilarity between bacterial profiles was visually assessed through principal coordinates analysis (PCoA). A Venn diagram was also built to visualize the general taxonomic differences between fish groups. ASVs were collapsed to both phyla and genera, and taxa with relative abundance < 1% were filtered out. The relative abundance of the most represented taxa ( > 1%) was calculated per sample, and was then compared between groups using linear models, followed by pairwise t-tests with Bonferroni corrections for multiple comparisons when significant differences were found.

#### 2.4.2 Analysis of bacterial function

Predicted bacterial metabolic functions were estimated using the non-normalized ASV table and metagenomic Phylogenetic Investigation of Communities by Reconstruction of Unobserved States software (PICRUSt2). The function picrust2_pipeline.py was used with default settings (e.g. normalisation by predicted 16S copy number abundances and max NSTI ≥ 2) that produces (1) sequence placement, (2) hidden-state prediction of genomes, (3) metagenome prediction, and (4) pathway-level predictions (Douglas et al., 2020). The software ggpicrust2 (Yang et al., 2023) was used to convert the Kyoto Encyclopedia of Genes and Genomes (KEGG) Orthology (KO) abundance table to KEGG pathway abundance (Kanehisa et al., 2019). Pathway differential abundance analysis (DAA) between fish groups was performed using DESeq2 (Love et al., 2014), as implemented in ggpicrust2. Principal Component Analysis (PCA) on functional pathway abundance data was performed to depict the differences, after dimensional reduction, between asymptomatic and symptomatic groups.

## 3 Results

### 3.1 Differences in bacterial diversity

**FIGURE 1:**
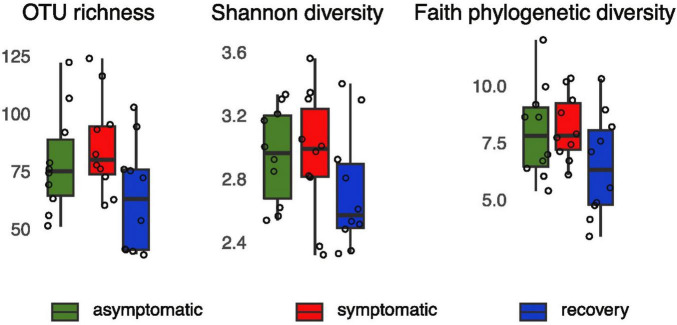
OTU richness, Shannon and Faith’s phylogenetic PD of asymptomatic, symptomatic and recovery fish groups based on 16S rRNA profiles.

**FIGURE 2:**
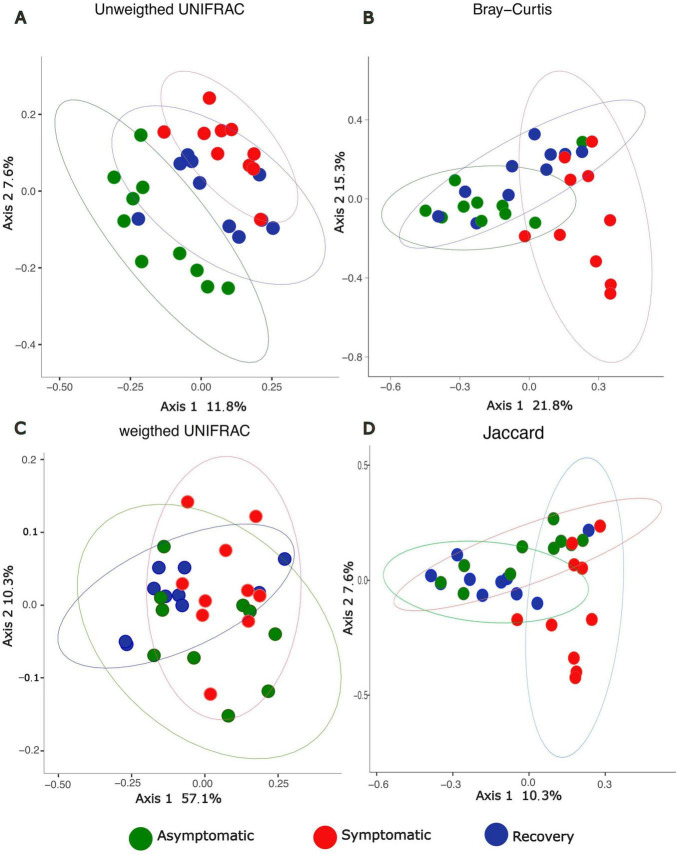
PCoA depicting beta-diversity dissimilarities based on 16S rRNA profiles between asymptomatic, symptomatic and recovery fish groups using **(A)** unweighted UNIFRAC, **(B)** Bray-Curtis, **(C)** Weighted UNIFRAC, and **(D)** Jaccard distances.

Alpha-diversity was lower in the recovery fish group, however the differences between groups were not statistically significant (1.34 < F < 2.62, *p* > 0.05, Figure 1). Analysis of beta-diversity with the function adonis2 showed significant differences between groups when considering the Jaccard, Bray-Curtis, and unweighted UNIFRAC distances (R^2^ = 0.11, 0.18, and 0.12, respectively; *p* < 0.05, Figure 2). However, dissimilarity between groups was not significant when weighted UNIFRAC distance was used (R^2^ = 0.13, *p* = 0.07, Figure 2). The function pairwise.adonis2 applied to unweighted UNIFRAC distances showed that all pairwise group comparisons were significant (Supplementary Table 1). However, when considering the Jaccard and Bray-Curtis distances only differences between asymptomatic and symptomatic, and between symptomatic and recovery groups were significant (Supplementary Table 1).

**FIGURE 3:**
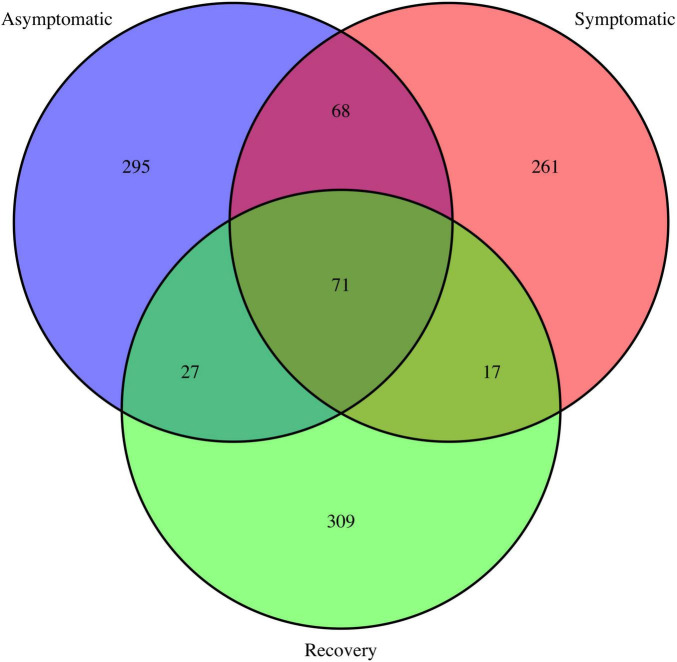
Venn diagram depicting shared and unique ASVs in the asymptomatic, symptomatic and recovery groups.

**FIGURE 4:**
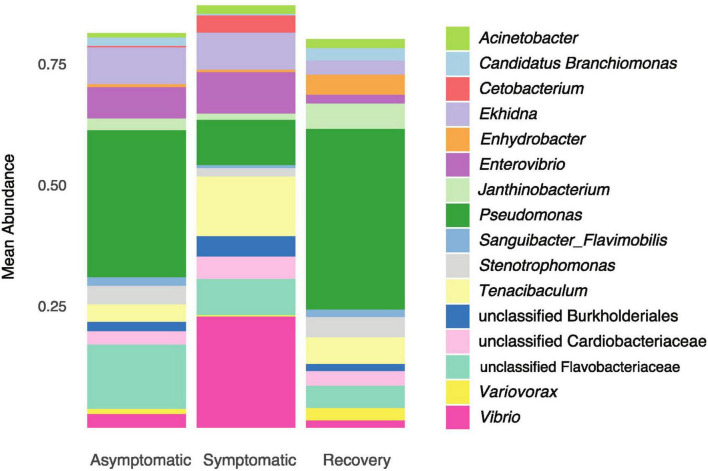
Mean relative abundance of the dominant genera (>1%) in asymptomatic, symptomatic and recovery groups.

The Venn diagram showed most ASVs are exclusive to each fish group, with only 71 ASVs being shared between all fish groups (Figure 3). The asymptomatic and symptomatic group shared 68 ASVs, whereas the symptomatic and recovery groups shared 17 ASVs, with this number increasing to 27 between the recovery and asymptomatic groups (Figure 3). Most taxa on the skin of the analysed fish belonged to phyla Proteobacteria (average 69%), followed by Bacteroidota (average 24%). Three other phyla had relative abundances ≥ 1%: Actinobacteria (2.7%), Fusobacteria (1.3 %), and Firmicutes (1%). There were no significant differences between these phyla between fish groups (Supplementary Figure 2). Among the most abundant genera, only *Enterovibrio, Vibrio, Pseudomonas*, and *Variovorax* varied significantly between fish groups (Figure 4 and Supplementary Table 2). *Enterovibrio* decreased and *Variovorax* increased in the recovery group relative to the symptomatic group. *Vibrio* was more abundant in fish showing LVD symptoms compared to asymptomatic and recovery groups. Conversely, *Pseudomonas* was more abundant in asymptomatic and recovery fish groups. *Tenacibaculum* was also much more abundant on symptomatic fish, albeit not significantly. Since one asymptomatic and one recovery sample showed an unexpectedly high abundance of *Tenacibaculum* spp. (Supplementary Figure 3), the interquartile range (IQR) method was used to determine whether samples were outliers. After confirming these were indeed statistical outliers, we removed them and re-calculated the differences in the abundance of *Tenacibaculum*, which showed significant differences in abundance between all groups (F = 8.64; *p* < 0.0015; Supplementary Table 2). According to comparisons against Silva and MiNT databases, 12 out of the 19 ASVs belonging to *Tenacibaculum* were taxonomically assigned to *Tenacibaculum maritimum* via phylogenetic analysis, a pathogen known to infect seabream (Avendaño-Herrera et al., 2006). Similarly, 9 out of 22 *Vibrio* ASVs were also identified as pathogens of aquatic invertebrates and fish: *Vibrio crassostreae, Vibrio mediterranei, Vibrio comitans, Vibrio fortis, and Vibrio ponticus*; however most ASVs did not attain species assignment in the MiMT database.

### 3.2 Differences in bacterial function between fish groups

**FIGURE 5:**
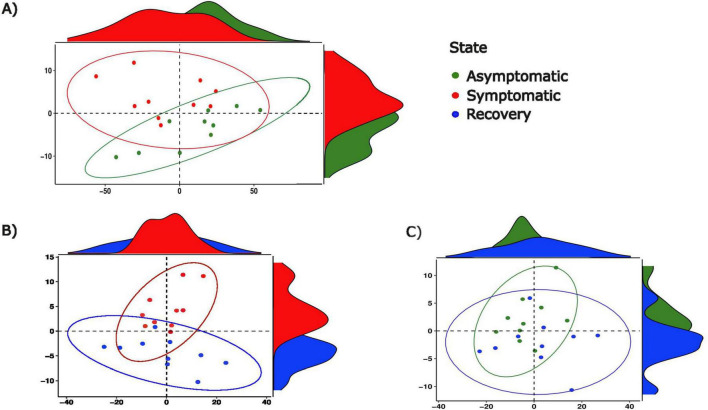
Principal component analyses of functional pathway abundance between **(A)** asymptomatic and symptomatic fish, between **(B)** symptomatic and recovery fish and, between **(C)** asymptomatic and recovery groups.

PCA depicted differences between the abundance of functional pathways between groups, with the symptomatic group differing from asymptomatic and recovery groups, and minimal differences existing between the latter two (Figures 5A–C). A total of 87 metabolic pathways were differentially enriched between asymptomatic and symptomatic fish, of which 84 were successfully annotated (Supplementary Table 3 and Supplementary Figure 4). From these, 56 pathways were related to metabolism with the majority of them involving amino acids (*n* = 7), lipids (*n* = 8) and carbohydrates (*n* = 9) and the biodegradation of xenobiotics (*n* = 12). In the case of the metabolism of xenobiotics, nine pathways were enriched in asymptomatic fish relative to symptomatic fish. Although the differences between symptomatic and recovery groups were also large (119 differentially abundant functional pathways), the differences between asymptomatic and recovery group were limited to a single pathway related to the endocrine system (peroxisome proliferator-activated receptors (PPARs) signalling pathway; Supplementary Table 2).

## 4 Discussion

We investigated the impact of LVD in the skin bacteriome of gilthead seabream juveniles reared in a single tank by examining the differences in diversity and predicted function between symptomatic, asymptomatic and recovered fish. Our results showed that LVD significantly impacts the bacteriome of fish, with dysbiosis occurring through alterations of bacterial community structure and imbalance persisting in recovered fish. Importantly, an increase of bacterial pathogens known to impact gilthead seabream health was also observed. Strikingly, less than 10% of all ASVs were common to asymptomatic, diseased and recovered fish. Although this might also be a result of our low sample size, it agrees well with the previously reported dynamic nature of fish skin microbiota (e.g. Rosado et al., 2021; Berggren et al., 2022) and how it may be affected by fish homeostasis (Xavier et al., 2024).

Proteobacteria and Bacteroidota were the most abundant phyla in all fish analysed. This result is in agreement with those reported in previous studies of the skin microbiota of healthy and diseased gilthead seabream fingerlings farmed in Portugal (Rosado et al., 2023; Rosado et al., 2021). Changes in the abundance of pivotal phyla in the gut of vertebrates, specifically of Proteobacteria:Bacteroidota (P:B) or Proteobacteria:Firmicutes (P:F) ratios, have been linked to disease (e.g. Zhang et al., 2016). In fish, changes to P:B/P:F ratios were also found to occur in both gut and skin (Tran et al., 2018; Ma et al., 2019; Cámara-Ruiz et al., 2021; Rosado et al., 2022). Indeed, a review of fish microbiome literature dealing with dysbiosis caused by infectious disease showed that changes of P:B/P:F ratios frequently occur in the skin of diseased fish (Xavier et al., 2024). However, in the present study, no significant changes in the abundance of these three phyla were found.

We observed significant changes in the mean relative abundance of some of the main bacterial genera; *Tenacibaculum maritimum* and *Vibrio* pathogens increased in symptomatic fish. *Tenacibaculum* and *Vibrio* spp. also increased their abundance in the skin of gilthead seabream suffering from photobacteriosis, indicating that disease-elicited skin microbial imbalance creates favourable conditions for proliferation of these specific opportunistic pathogens (Rosado et al., 2023). In the specific case of *Tenacibaculum maritimum*, it was suggested that interspecific interactions with other bacterioplankton and microorganisms (e.g., bacteriophages) present in seawater is one of the mechanisms that regulates this pathogen’s abundance (reviewed in Avendaño-Herrera et al., 2006).

Comparisons of the predicted microbiome function between diseased and asymptomatic fish showed that the changes in microbiome structure translated into a decrease in the metabolic ability to degrade xenobiotics in diseased fish. Biodegration of xenobiotics is one important role that bacteria play in the aquatic ecosystems environment through which pollutant substances are transformed in harmless forms (Rahman et al., 2024). Likewise, bacterial commensals that degrade these toxic pollutants can have an important detoxifying role on host tissues (e.g. Hernández-Mendoza et al., 2022; Mendoza et al., 2018). For example, bacteria that degrade xenobiotics have been positively associated with extreme longevity in humans (Rampelli et al., 2020). Although analysis of changes in microbiome function caused by dysbiosis in aquaculture settings has not been the focus of much research (reviewed by Xavier et al., 2024), biodegradation of xenobiotics by bacteria in fish farms was sometimes found to be enriched during unfavourable periods. For example, increased microbial degradation of xenobiotics has been hypothesized to be a response to changes in water quality in aquaculture systems that ultimately resulted in dysbiosis in the external mucosae of Atlantic salmon (*Salmo salar*) (Lorgen-Ritchie et al., 2022). Additionally, in farmed catfish (*Heteropneustes fossilis*) and grass carp (*Ctenopharyngodon idellus*), xenobiotic biodegradation was enriched in diseased fish (Tran et al., 2018; Sultana et al., 2022). Conversely, in the present study, most metabolic pathways for xenobiotic biodegradation were suppressed in symptomatic fish, i.e. during dysbiosis. This suggests that dysbiosis caused by LVD negatively affected the protective role of the skin microbiota. Functional differences between the recovery and asymptomatic groups were restricted to a single pathway, indicating that microbiome functionality was mostly fully recovered in the three weeks following the appearance of symptoms. Again, a similar result was observed in dysbiotic bacterial communities from the skin of gilthead seabreams suffering from photobacteriosis, where metabolic pathways of detoxification were enriched in asymptomatic and recovered individuals when compared to diseased individuals (Rosado et al., 2023). *Pseudomonas* and *Variovorax* strains are among the main bacterial groups known to degrade xenobiotics (e.g. Ghimire et al., 2022; Akkaya et al., 2018); since the abundance of these bacteria was significantly increased in asymptomatic and recovered fish, we can hypothesize that they were responsible for the xenobiotic biodegradation predicted to occur in microbial communities of these fish.

Despite the dysbiosis observed in diseased fish, the diversity and functionality of the skin microbiome was mostly recovered after three weeks, following a 2-fold decrease in fish density. Asymptomatic and recovery groups showed unique bacterial communities, as suggested by the unweighted phylogenetic distances between their taxa. However, Bray-Curtis distances indicated that the recovery group has regain bacterial community structure, and predicted microbiome function suggested a high degree of functional redundancy despite the phylogenetic uniqueness of each community. Even the mean relative abundance of both pathogenic taxa (*Tenacibaculum* and *Vibrio*) and those potentially linked to biodegradation of xenobiotics (*Pseudomonas* and *Variovorax*) were similar between asymptomatic and recovery groups.

## 5 Conclusion

We have shown that LVD has a severe effect on the skin microbiome of gilthead seabream, whereby changes in bacterial community structure also lead to changes in their functionality. The present results show that LVD elicited microbial changes that led to an “unhealthy” bacteriome, where fish pathogens such as *Tenacibaculum maritimum* and several *Vibrio* spp have emerged. This microbial imbalance may turn this usually benign and self-limited viral infection into a secondary severe infection that causes fish mortality. Reducing fish tank density for three weeks helped the skin microbiome to revert to a healthier state; a smaller tank population could result in lesser fish stress and lower bacterial transmission among individuals, consequently leading to a rapid improvement of host homeostasis after LVD.

## Data Availability

The metagenomic data presented in the study are deposited in the SRA repository of NCBI under the project PRJNA1167648 (https://www.ncbi.nlm.nih.gov/sra/PRJNA1167648).
