# Supplementary figures and images for: Lymphocystis viral disease impacts the diversity and functional profiles of the skin microbiome in gilthead seabream
Source: Front Microbiol. 2024 Oct 21;15:1470572. doi: 10.3389/fmicb.2024.1470572 (PMC11532066; doi:10.3389/fmicb.2024.1470572)

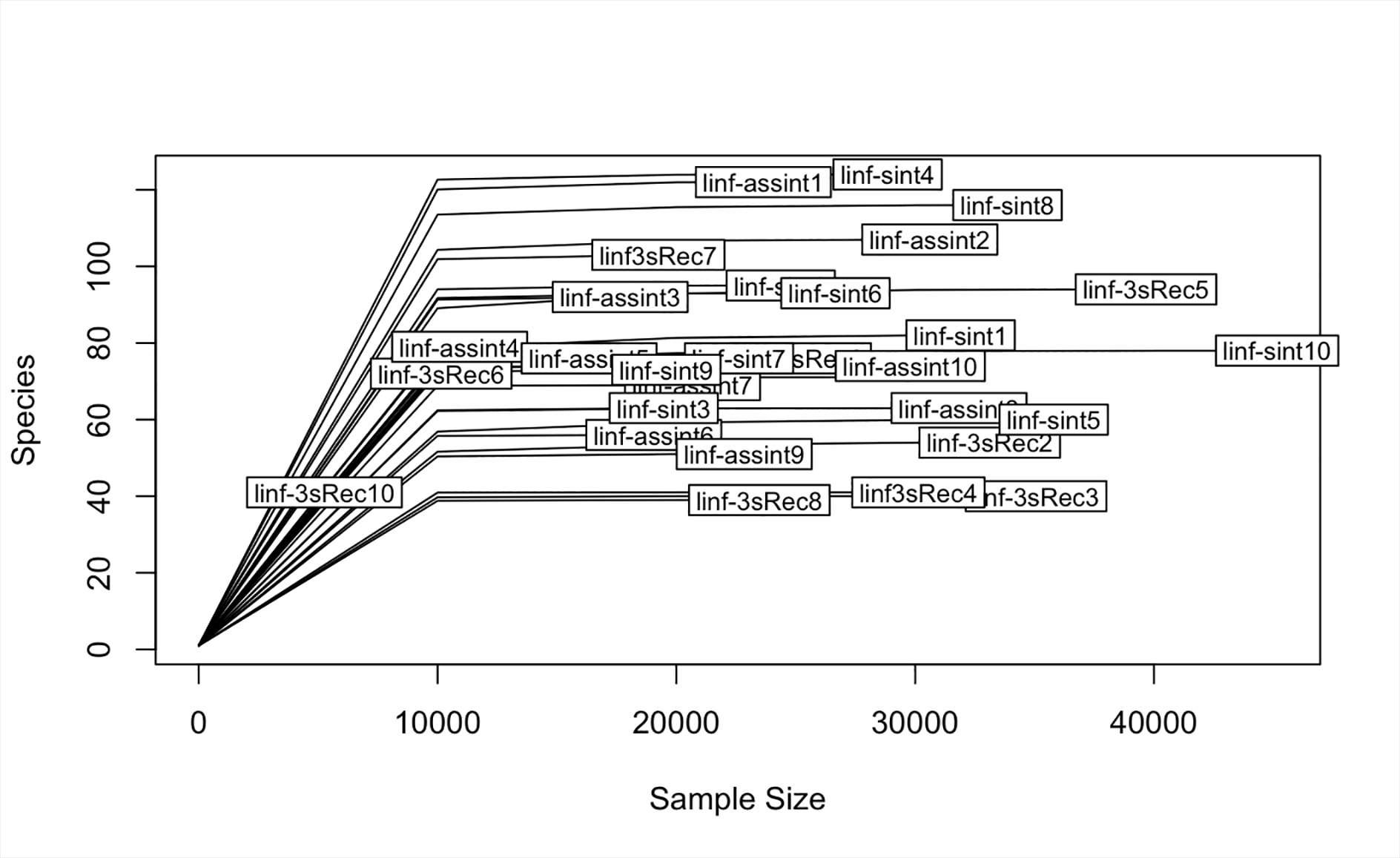

Supplement: Supplementary file 2 [file Image_1.TIFF]

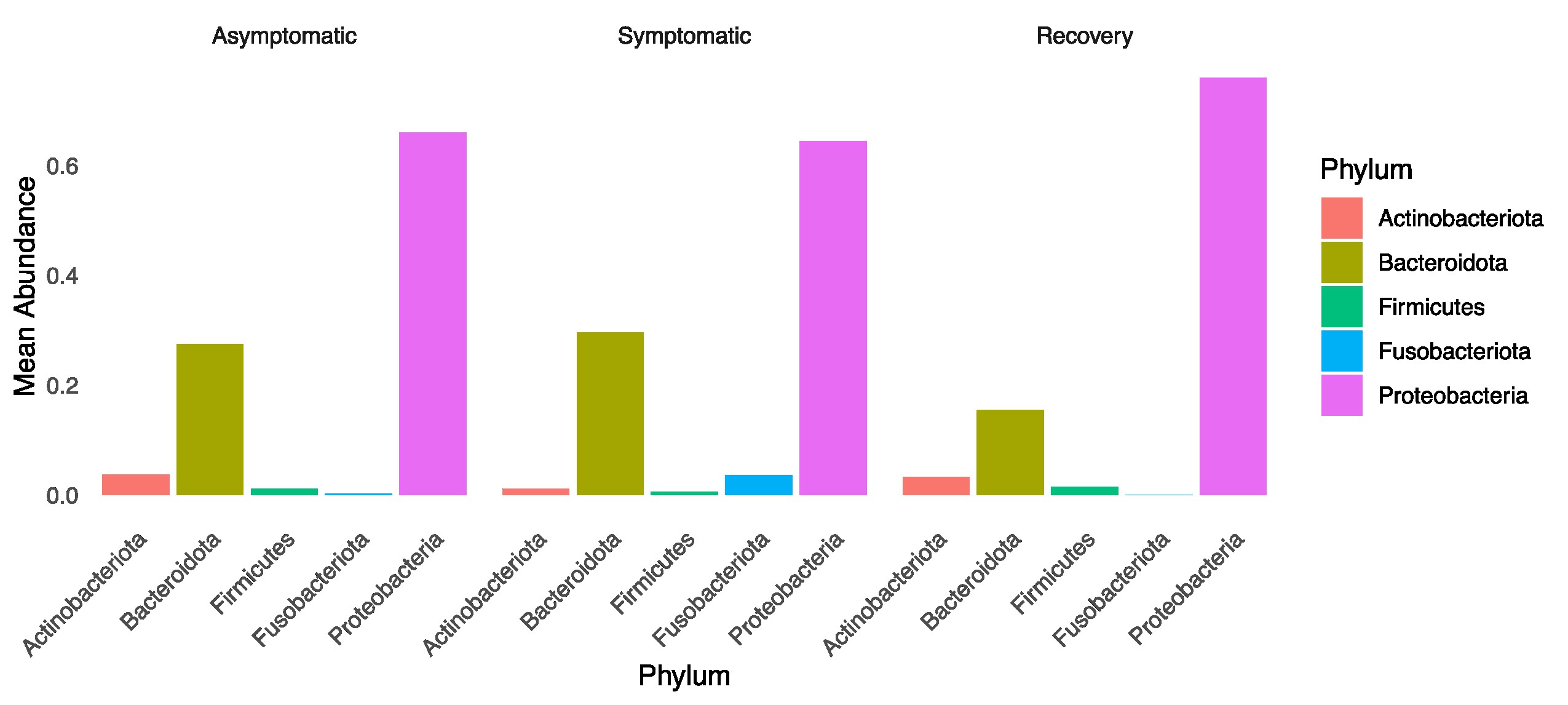

Supplement: Supplementary file 3 [file Image_2.JPEG]

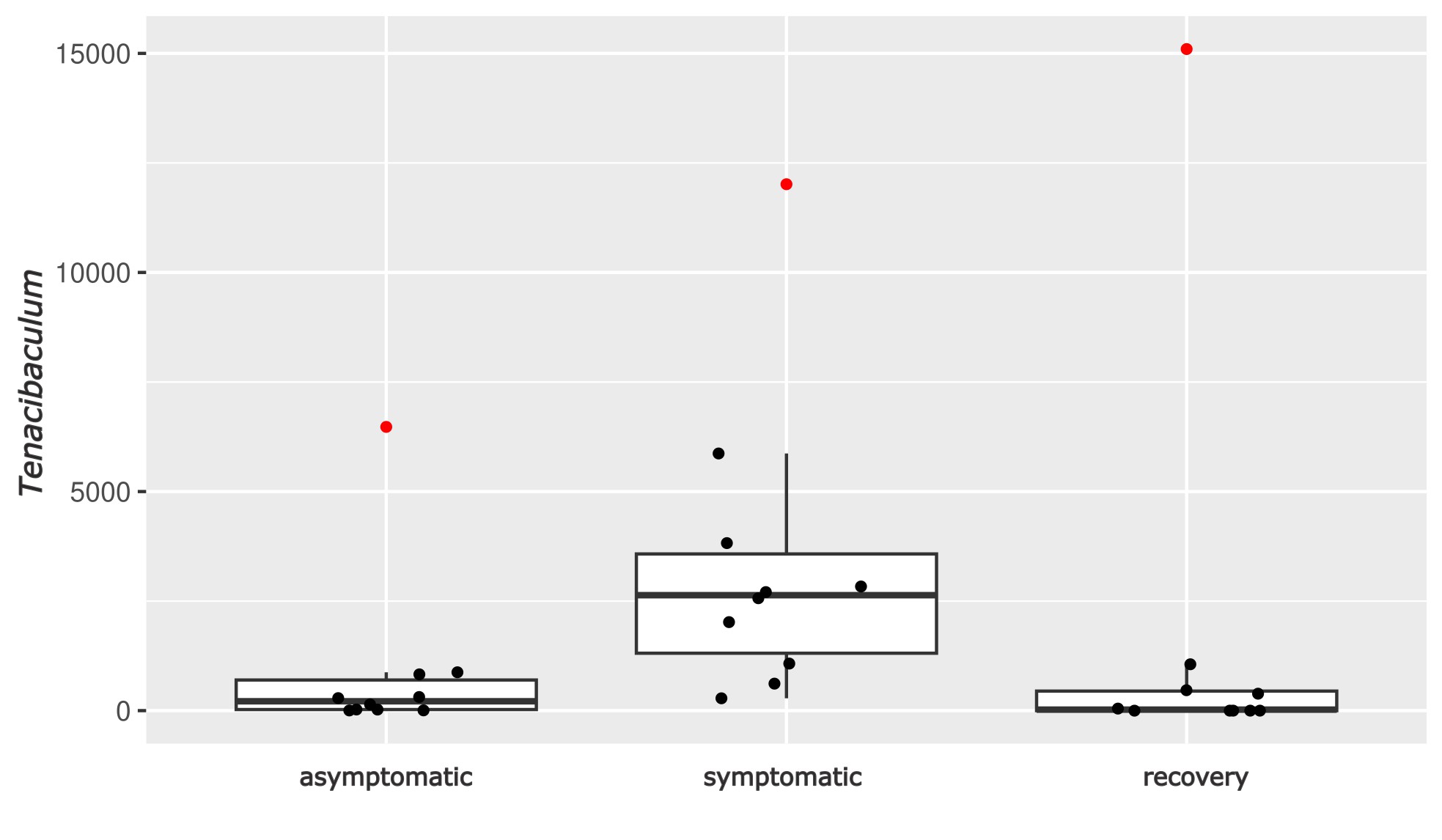

Supplement: Supplementary file 4 [file Image_3.JPEG]

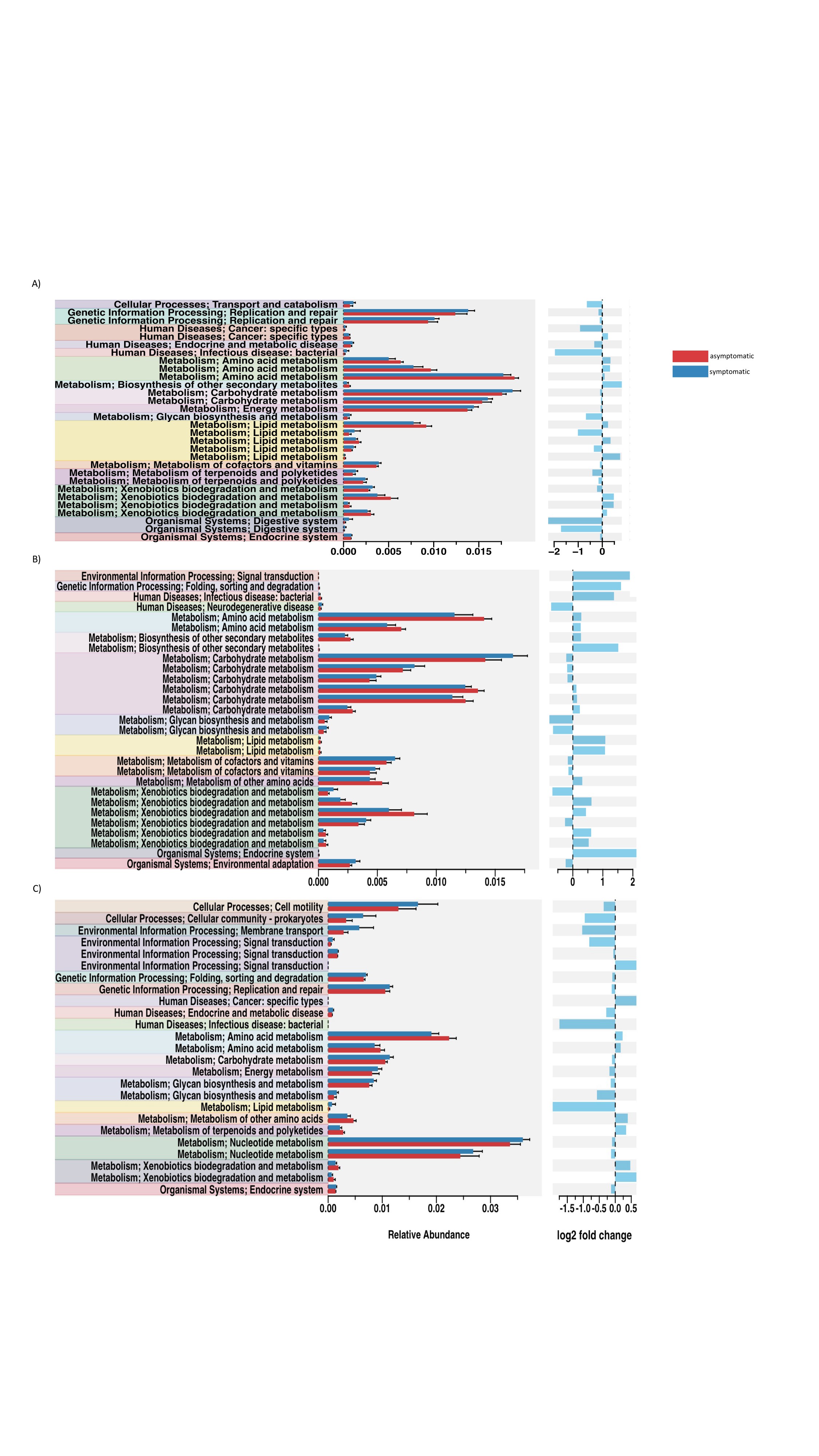

Supplement: Supplementary file 5 [file Image_4.JPEG]
